# Supplementary material for: Three-Dimensional Trilineage Differentiation Conditions for Human Induced Pluripotent Stem Cells
Source: Bioengineering (Basel). 2025 May 9;12(5):503. doi: 10.3390/bioengineering12050503 (PMC12108907; doi:10.3390/bioengineering12050503)
Supplement: Supplementary file 1 [file bioengineering-12-00503-s001.zip › bioengineering-3509427-supplementary.pdf]

bioengineering

MDPI

Supplementary Data

Table S1. Real-time quantitative PCR (RT-qPCR) primers.

Gene markers	Primer ID	Sequence	Tm(°C)	
OCT4	OCT4-F1	AAAGAGAAAGCGAACCAG	49.9	52
	OCT4-R1	CCACATCCTTCTCGAGCC	55.3	
FOXA2	FOXA2-F	AGATGGAAGGGCACGAGC	61.35	63
	FOXA2-R	CAGGCCGGCGTTCATGTT	65.51	
Brachyury	Brac-F	GAACGAGGAGATCACAGCTCTT	60.2	60
	Brac-R	TTCCTCCATCATCTCTTTGTGA	59.8	
PAX6	PAX6-F3	TGCAGATGCAAAAGTCCAAG	59.99	60
	PAX6-R3	TGATGGAGCCAGTCTCGTAA	59.39	
hEID2*	hEID2-F	GAAGCCTGCAGAGCAAGG	59.8	60
	hEID2-R	ATATCGAGGTCCACCCTGTG	59.8	
hZNF324B*	hZNF324B-F	GAGAATGGCCACGAGCTTT	60.4	60
	hZNF324B-R	TTTACACTGTGGCAGGCATC	59.7	

*Housekeeping gene

Table S2. A list of components and their specific volumes to prepare stained and isotype control samples

Component	Volume to add to the tube labeled	
	**Specific stain**	**Isotype control**
Permeabilized cells (at 1 x 10^7 cells per mL)	100 µL (1 x 10^6 cells)	100 µL (1 x 10^6 cells)
Endoderm differentiated cells		
Alexa Fluor 488 Mouse anti-Oct3/4 (BD Pharmingen, Catalog No: 560253)	20 µL	—
Human HNF-3 beta /FoxA2 Alexa Fluor® 647-conjugated (R&D system, Catalog: FAB24001R)	2.5 µL	-
Alexa Fluor 488 Mouse IgG1 κ isotype control (BD Pharmingen, Catalog No: 565572)	—	5 µL
Rabbit IgG Alexa Fluor® 647- isotype control (R&Dsystem, Catalog: IC1051R)	-	5 µL
Mesoderm differentiated cells		
Alexa Fluor 488 Mouse anti-Oct3/4	20 µL	—
Human Brachyury APC-conjugated (R&Dsystems, Catalog: IC2085A)	10 µL	-
Alexa Fluor 488 Mouse IgG1 κ isotype control	—	5 µL
Goat IgG APC isotype control (R&Dsystems , Catalog: IC108A)	-	10 µL
Ectoderm differentiated cells		
Alexa Fluor 488 Mouse anti-Oct3/4	20 µL	—
Alexa Fluor 647 Mouse Anti-human PAX6 (BD Pharmingen, Catalog No: 562249)	5 µL	—
Alexa Fluor 488 Mouse IgG1 κ isotype control	—	5 µL
Alexa Fluor 647 Mouse IgG2a, κ isotype control (BD Pharmingen, Catalog No: 558053)	—	20 µL

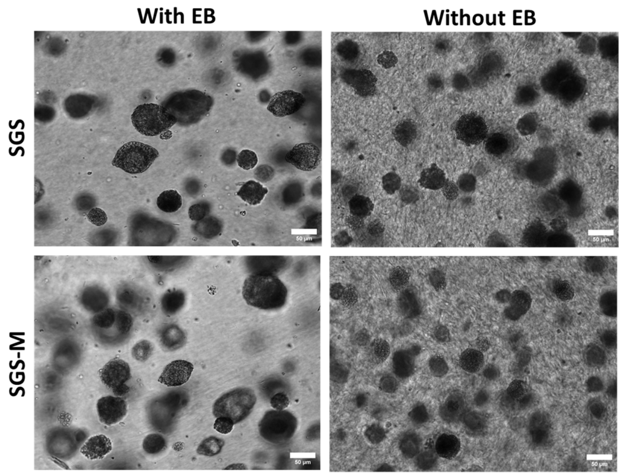
With EB
Without EB
SGS
SGS-M

(a)

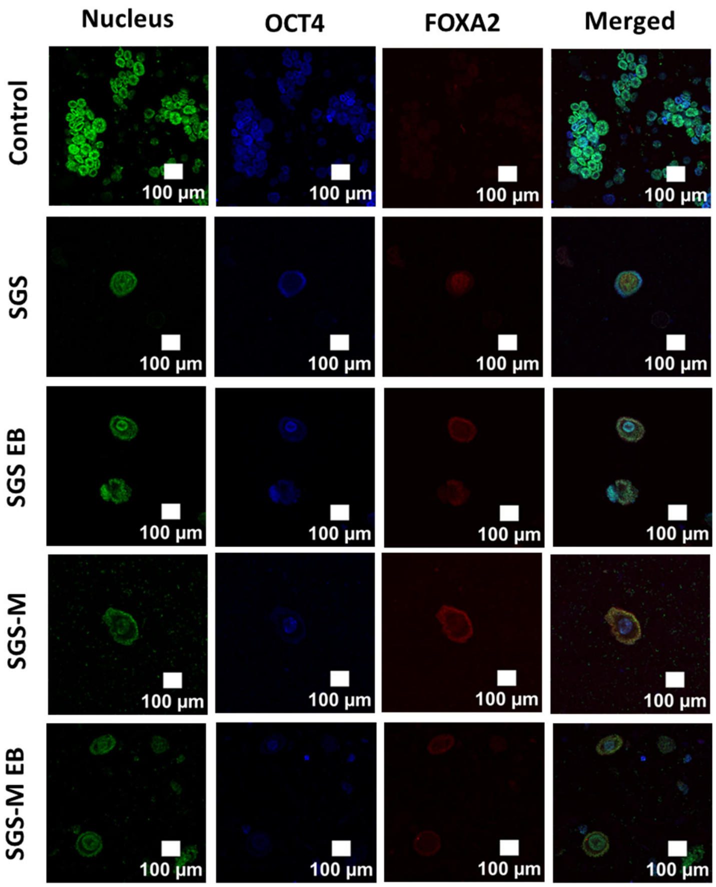
Nucleus
OCT4
FOXA2
Merged
Control
SGS
SGS EB
SGS-M
SGS-M EB
100 µm

(b)

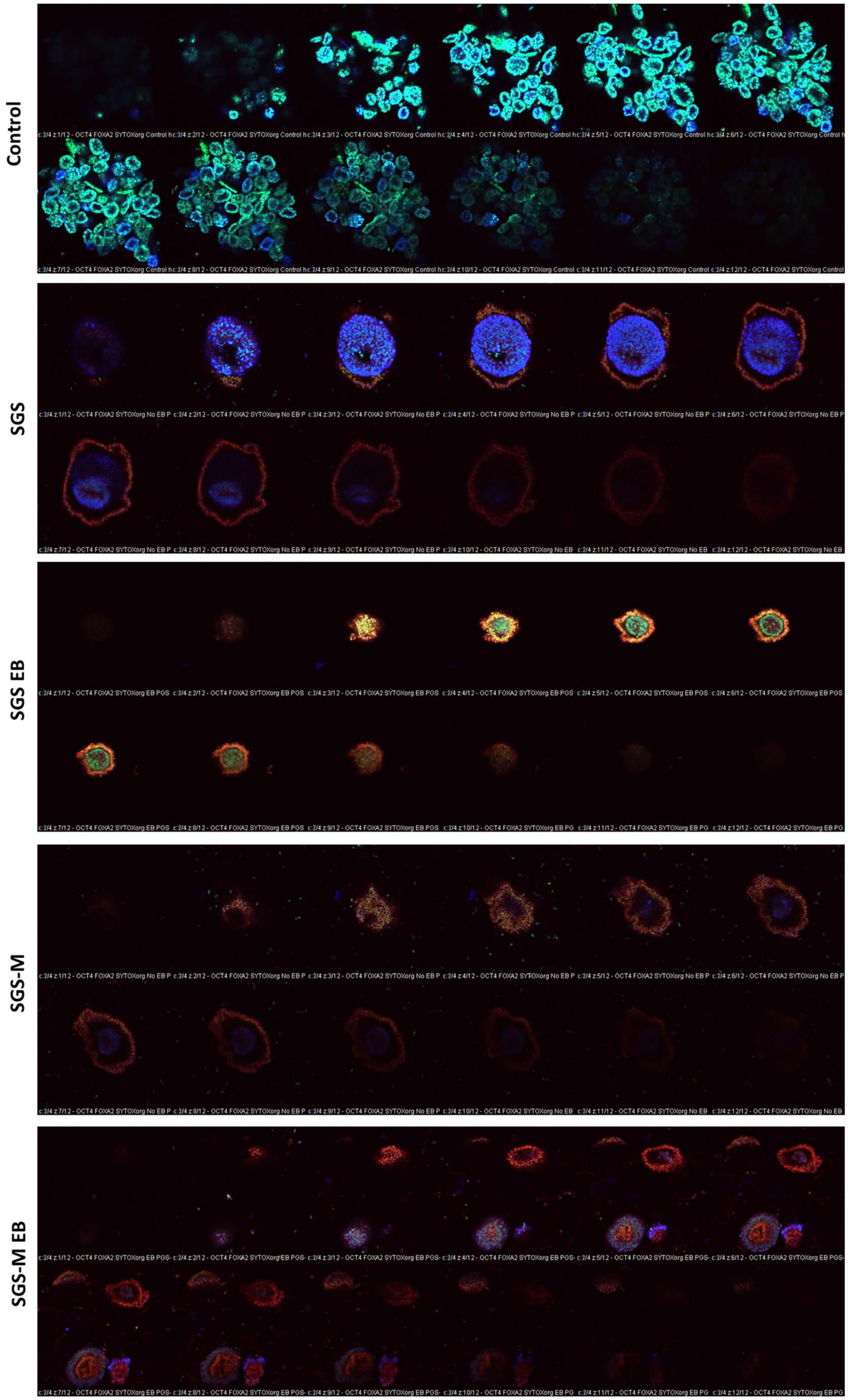

(c)

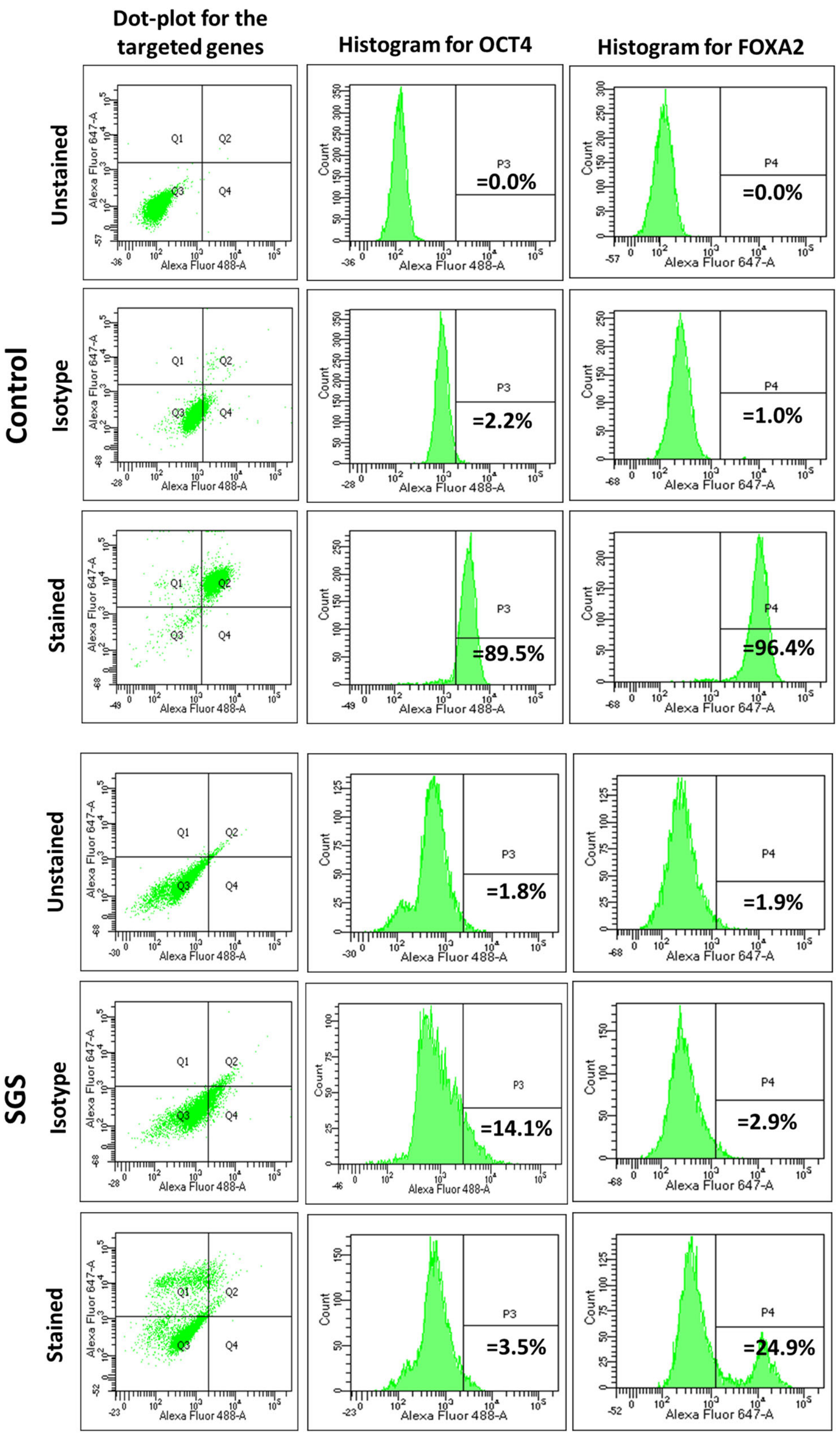

Dot-plot for the targeted genes
Histogram for OCT4
Histogram for FOXA2
Control
Unstained
Isotype
Stained
SGS
Unstained
Isotype
Stained
Alexa Fluor 647-A
Alexa Fluor 488-A
Count
Q1
Q2
Q3
Q4
P3
P4
=0.0%
=0.0%
=2.2%
=1.0%
=89.5%
=96.4%
=1.8%
=1.9%
=14.1%
=2.9%
=3.5%
=24.9%

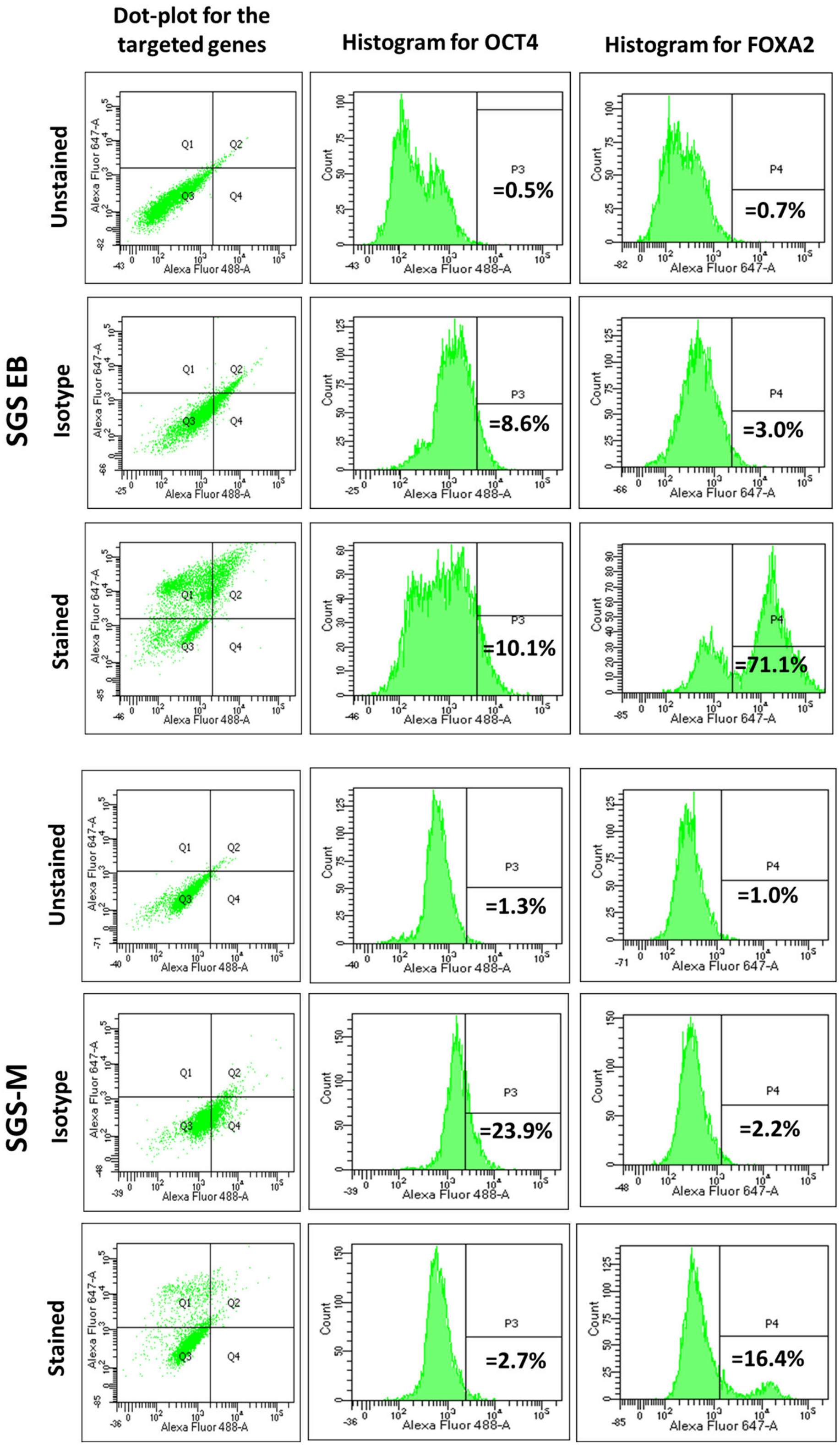
Dot-plot for the targeted genes
Histogram for OCT4
Histogram for FOXA2
SGS EB
Unstained
Isotype
Stained
SGS-M
Unstained
Isotype
Stained
Q1
Q2
Q3
Q4
P3
P4
Alexa Fluor 647-A
Alexa Fluor 488-A
Count
=0.5%
=0.7%
=8.6%
=3.0%
=10.1%
=71.1%
=1.3%
=1.0%
=23.9%
=2.2%
=2.7%
=16.4%

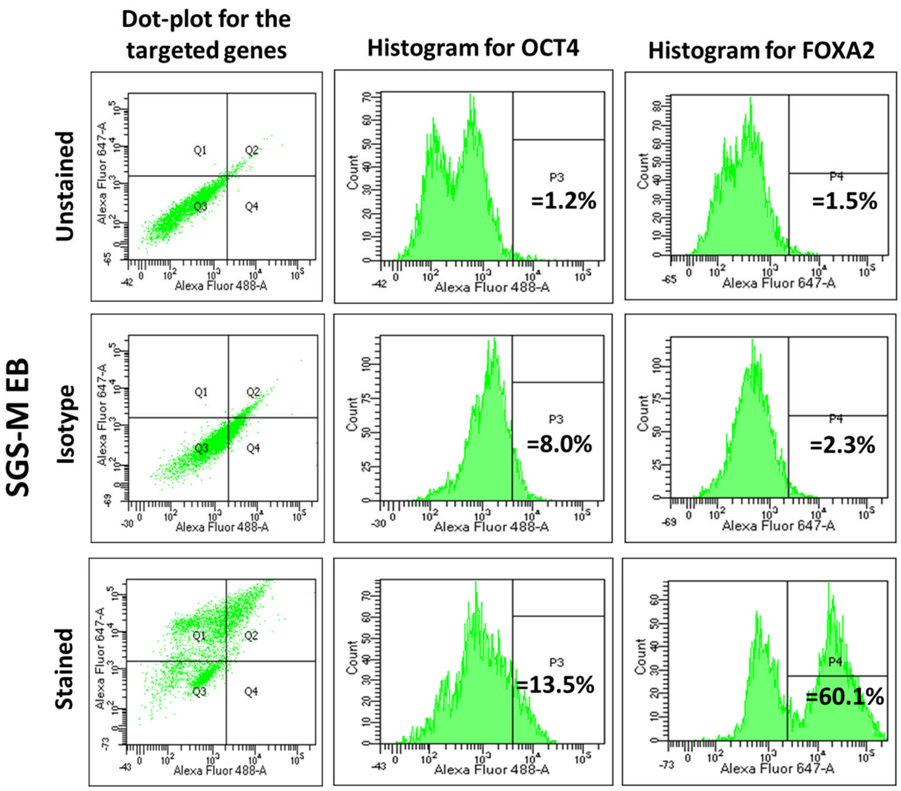

(d)

Figure S1. SGS matrix with EB medium induction determined a viable scaffold for endoderm differentiation of hiPSC spheroids. hiPSC was endoderm differentiated in 3D culture conditions in SGS, and SGS-M, with and without EB induction. Cells were harvested on day 5. (**a**) Cell morphology of endoderm differentiated hiPSC. The scale bar is 50 µm and the resolution is 10X; (**b**) Multichannel images of immunostained control and endoderm differentiated hiPSC. The cell was stained for nucleus, *OCT4*, and *FOXA2*. Nucleus, *OCT4*, and *FOXA2* were pseudo-colored with green, blue, and red respectively. The scale bar is 100 µm and the resolution is 10X; (**c**) Montage view of Z-stack multichannel images of immunostained control and endoderm differentiated hiPSC. Cells were stained for nucleus, *OCT4*, and *FOXA2*. Nucleus, *OCT4*, and *FOXA2* were pseudo-colored with green, blue, and red respectively. The images present merged channels. The resolution is 10X; (**d**) Flow cytometric analysis of control and endoderm differentiated hiPSC for *OCT4* and *FOXA2*. *OCT4* was labeled with AF488, and *FOXA2* was labeled with AF647 fluorochromes. Each sample was analyzed as unstained for autofluorescence, isotype for nonspecific binding, and stained for specific binding.

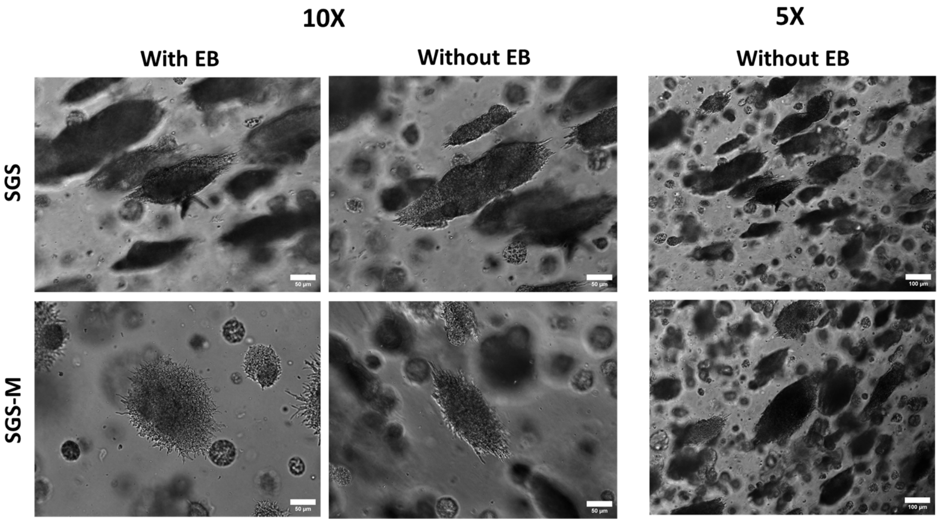
10X
5X
With EB
Without EB
Without EB
SGS
SGS-M

(a)

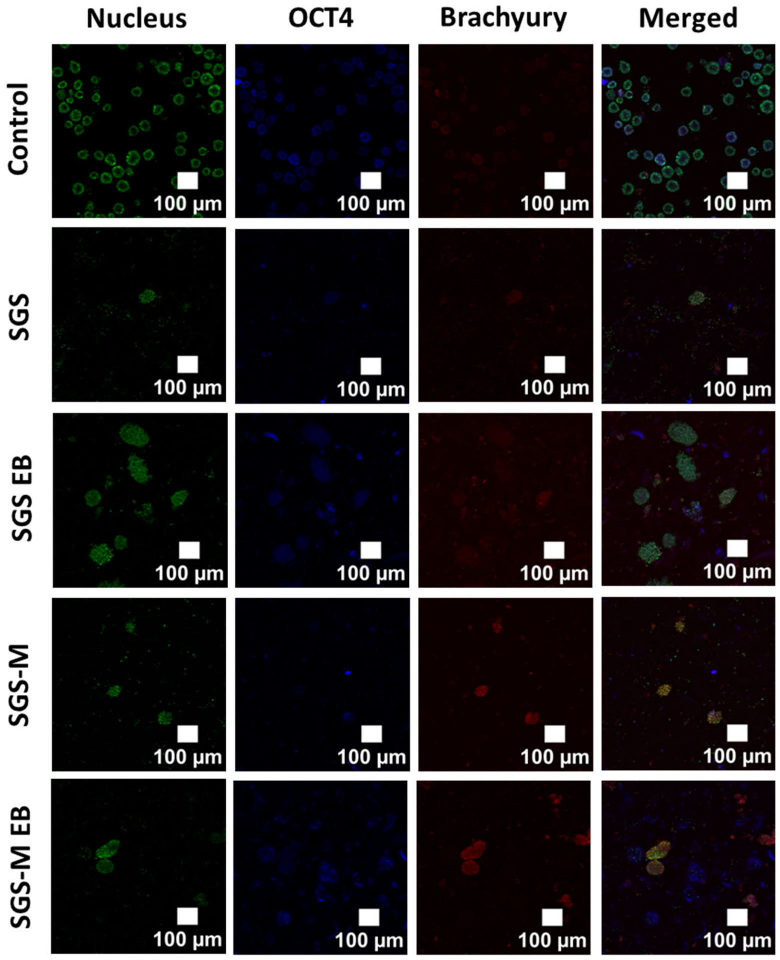
Nucleus
OCT4
Brachyury
Merged
Control
SGS
SGS EB
SGS-M
SGS-M EB
100 µm

(b)

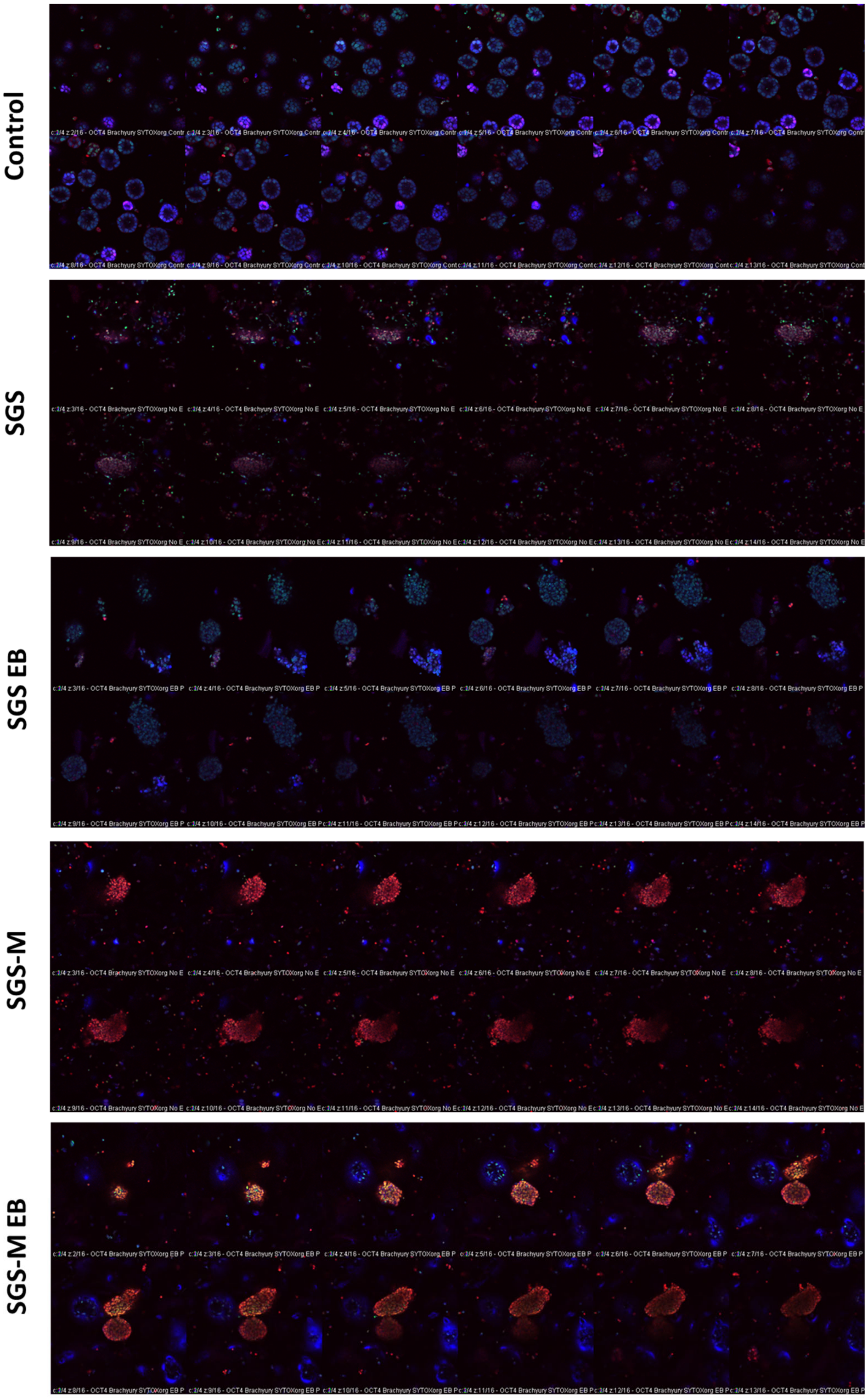

(c)

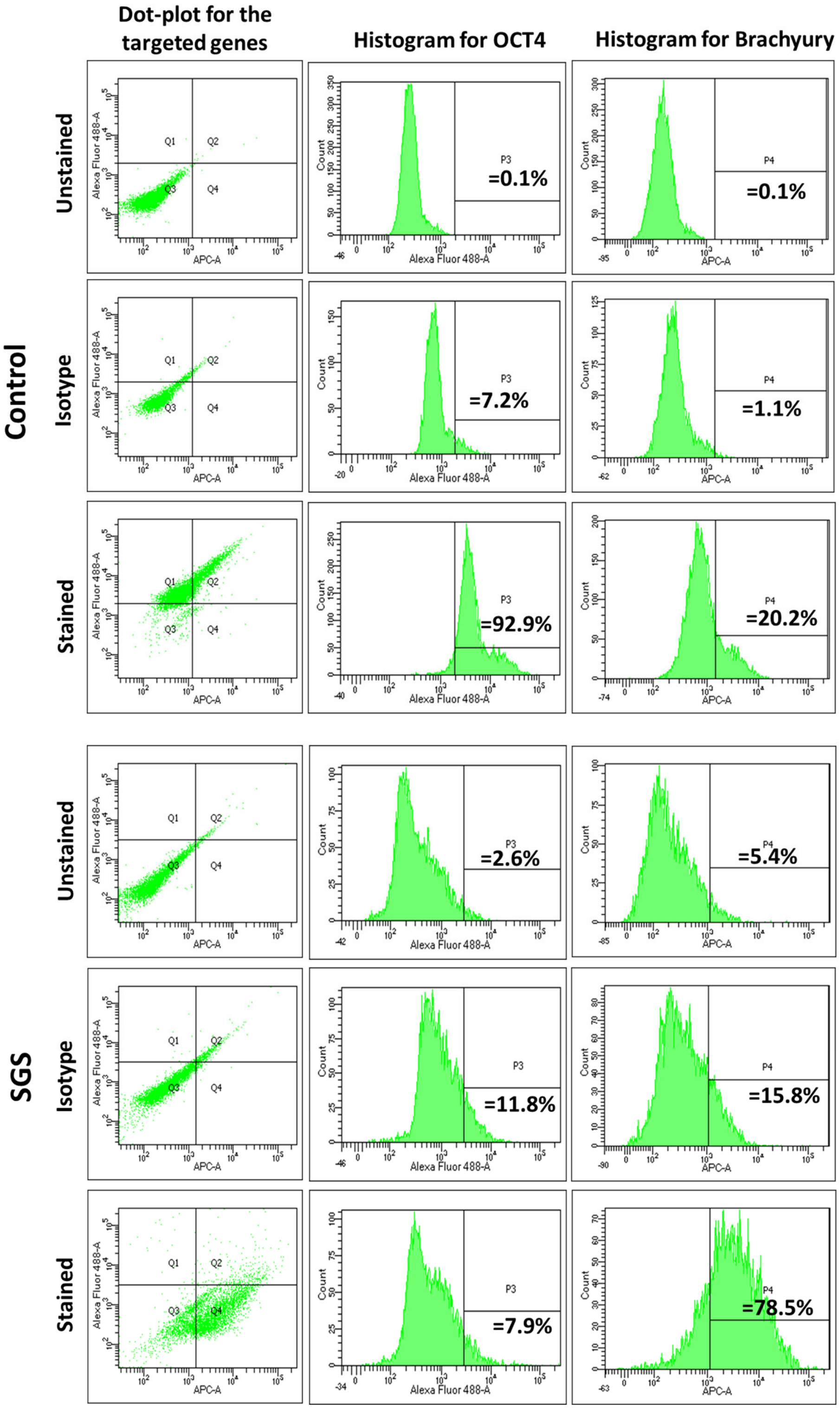

Dot-plot for the targeted genes
Histogram for OCT4
Histogram for Brachyury
Control
SGS
Unstained
Isotype
Stained
Unstained
Isotype
Stained
=0.1%
=0.1%
=7.2%
=1.1%
=92.9%
=20.2%
=2.6%
=5.4%
=11.8%
=15.8%
=7.9%
=78.5%

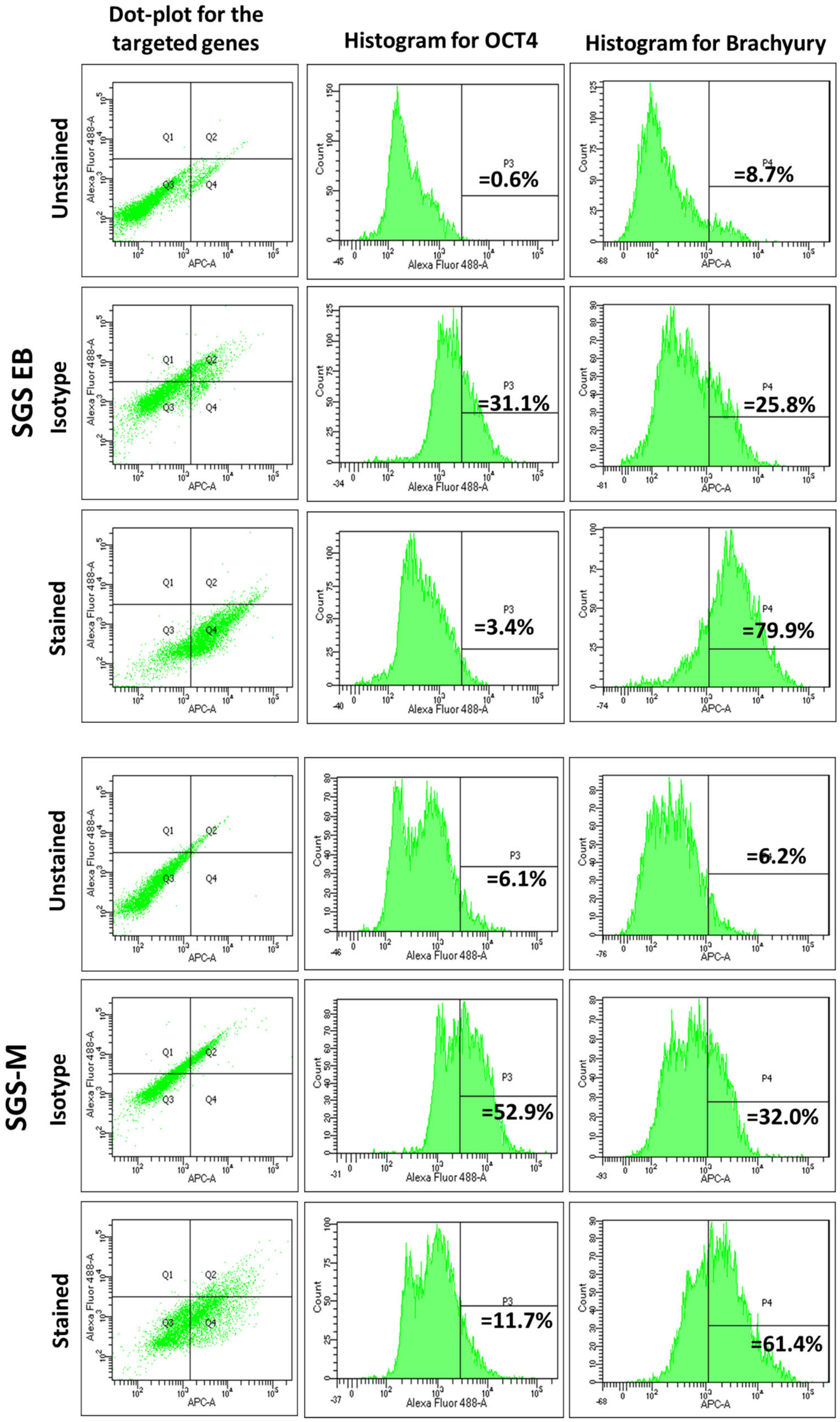

Dot-plot for the targeted genes
Histogram for OCT4
Histogram for Brachyury
SGS EB
SGS-M
Unstained
Isotype
Stained
Q1
Q2
Q3
Q4
APC-A
Alexa Fluor 488-A
Count
P3
P4
=0.6%
=8.7%
=31.1%
=25.8%
=3.4%
=79.9%
=6.1%
=6.2%
=52.9%
=32.0%
=11.7%
=61.4%

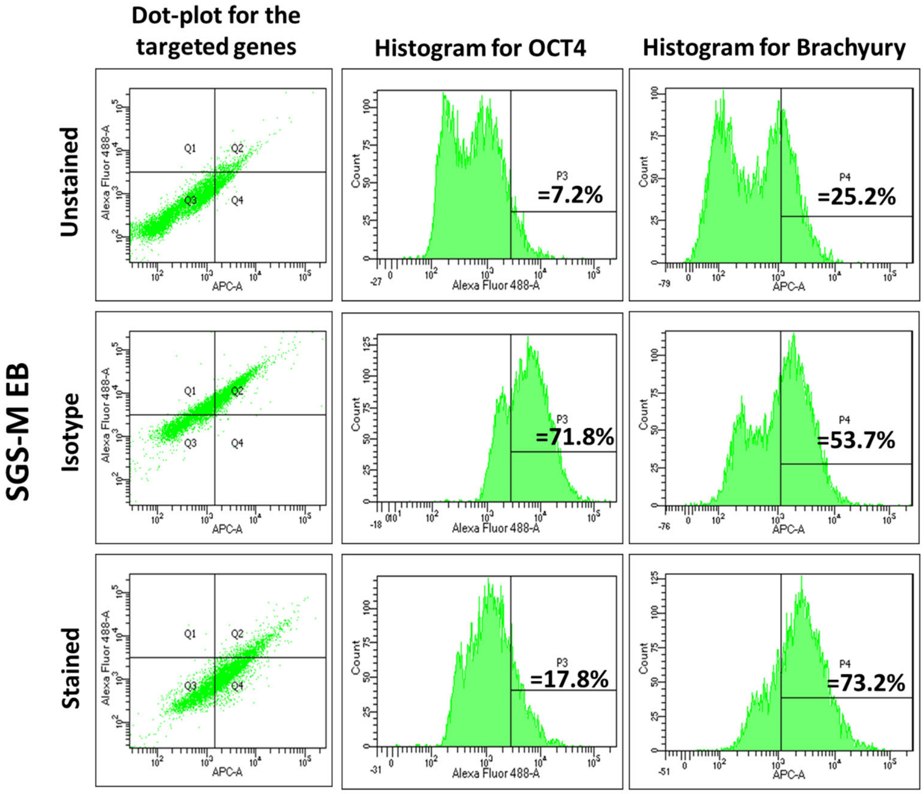

(d)

Figure S2. SGS matrix without EB induction presented an effective scaffold for mesoderm differentiation of hiPSC in 3D embedded condition. hiPSC was mesoderm differentiated in 3D culture condition in SGS, and SGS-M, with and without EB induction. Cells were harvested on day 5. (**a**) Cell morphology of mesoderm differentiated hiPSC. The scale bar is 50 μm for the images of 10X resolution and 100 μm for the images of 5X resolution; (**b**) Multichannel images of immunostained control and mesoderm differentiated hiPSC. Cells were stained for nucleus, *OCT4*, and *Brachyury*. Nucleus, *OCT4*, and *Brachyury* were pseudo-colored with green, blue, and red respectively. The scale bar is 100 μm and the resolution is 10X; (**c**) Montage view of Z-stack multichannel images of immunostained control and mesoderm differentiated hiPSC. Cells were stained for nucleus, *OCT4*, and *Brachyury*. Nucleus, *OCT4*, and *Brachyury* were pseudo-colored with green, blue, and red respectively. The images present merged channels. The resolution is 10X; (**d**) Flow cytometric analysis of control and mesoderm differentiated hiPSC for *OCT4* and *Brachyury*. *OCT4* was labeled with AF488, and *Brachyury* was labeled with APC fluorochromes. Each sample was analyzed as unstained for autofluorescence, isotype for nonspecific binding, and stained for specific binding.

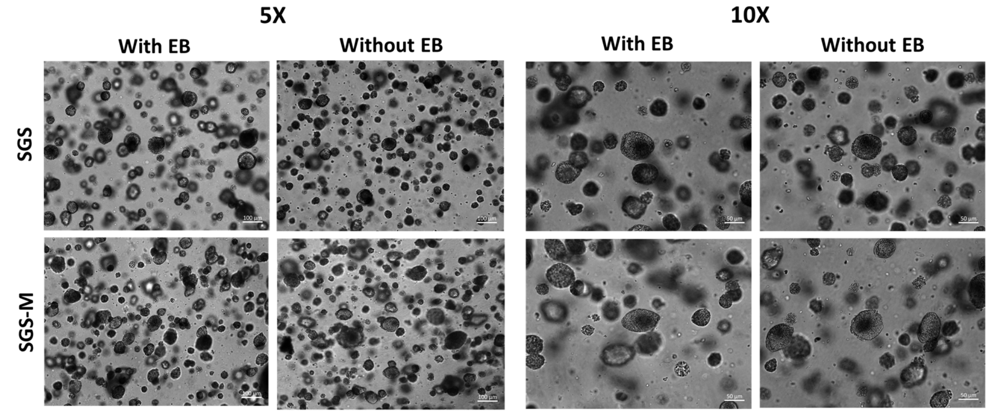
5X
With EB
Without EB
10X
With EB
Without EB
SGS
SGS-M

(a)

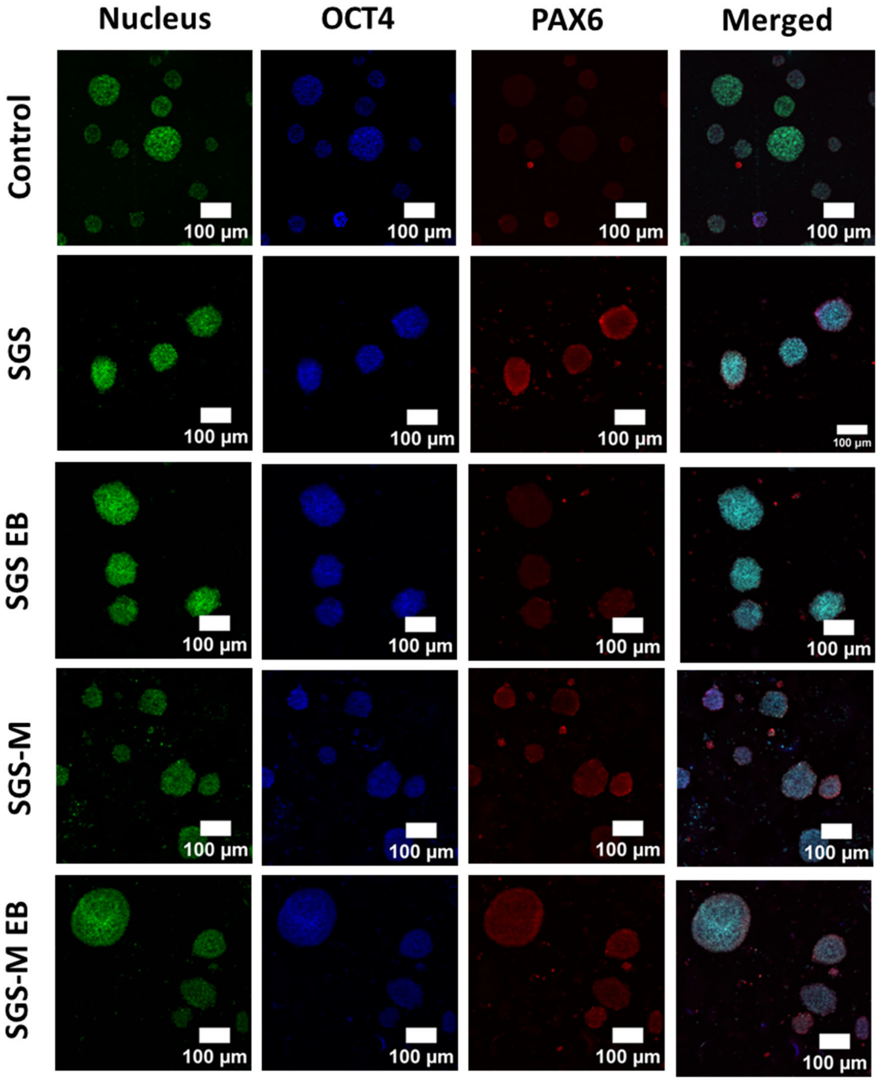
Nucleus
OCT4
PAX6
Merged
Control
SGS
SGS EB
SGS-M
SGS-M EB
100 µm

(b)

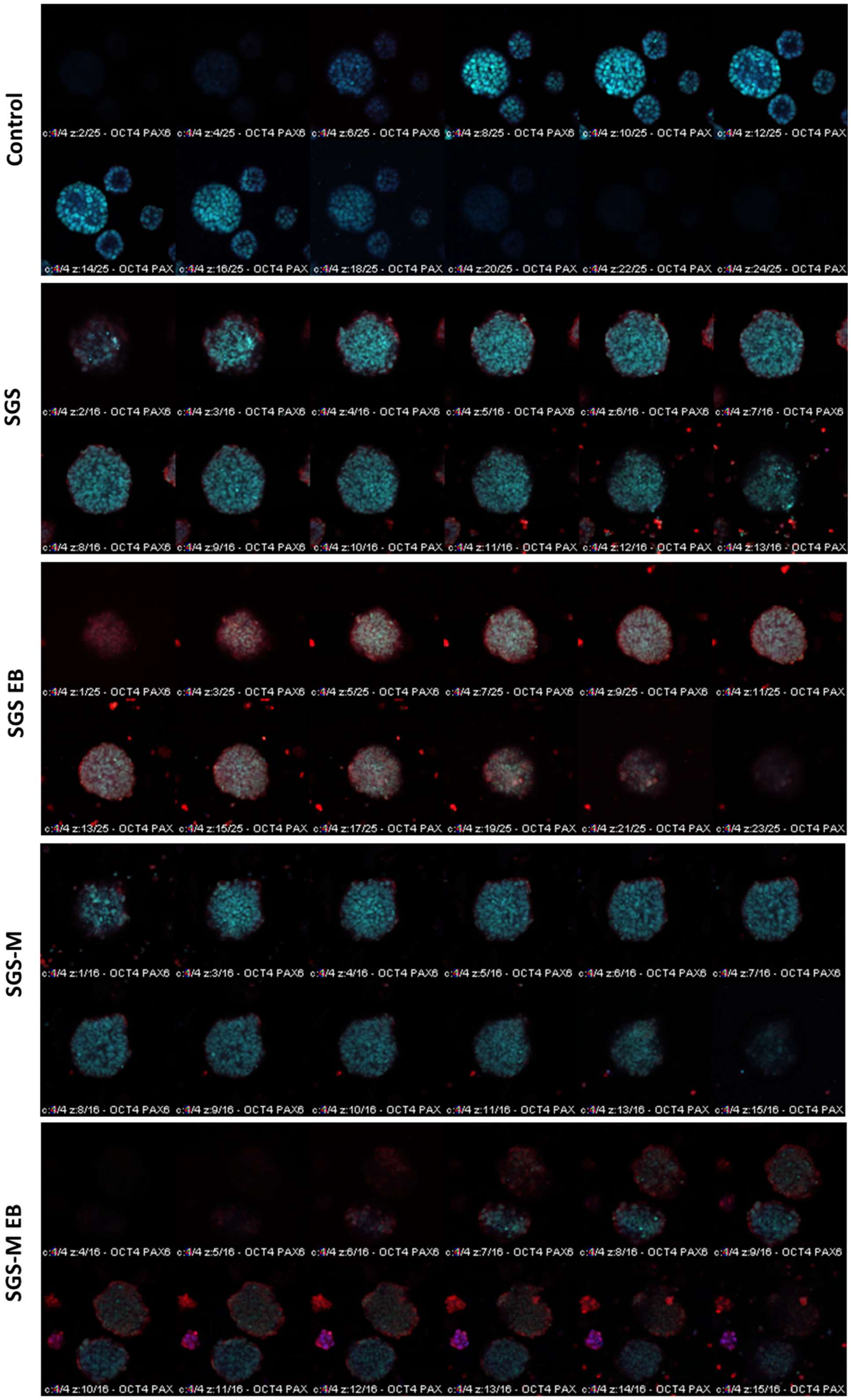

(c)

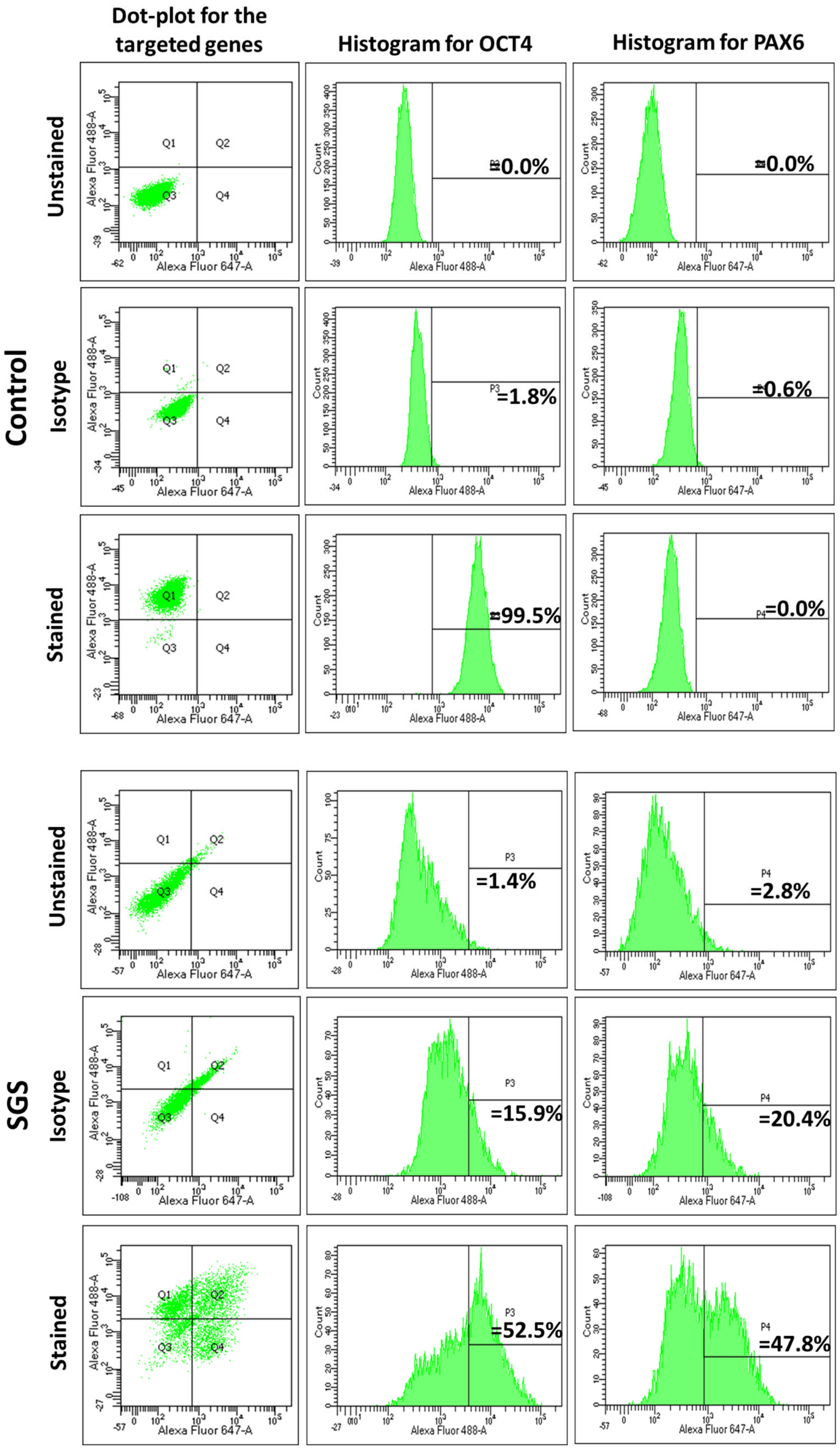

Dot-plot for the targeted genes
Histogram for OCT4
Histogram for PAX6
Control
Unstained
Isotype
Stained
SGS
Unstained
Isotype
Stained
Q1
Q2
Q3
Q4
Alexa Fluor 488-A
Alexa Fluor 647-A
Count
=0.0%
=0.0%
=1.8%
=0.6%
=99.5%
=0.0%
=1.4%
=2.8%
=15.9%
=20.4%
=52.5%
=47.8%

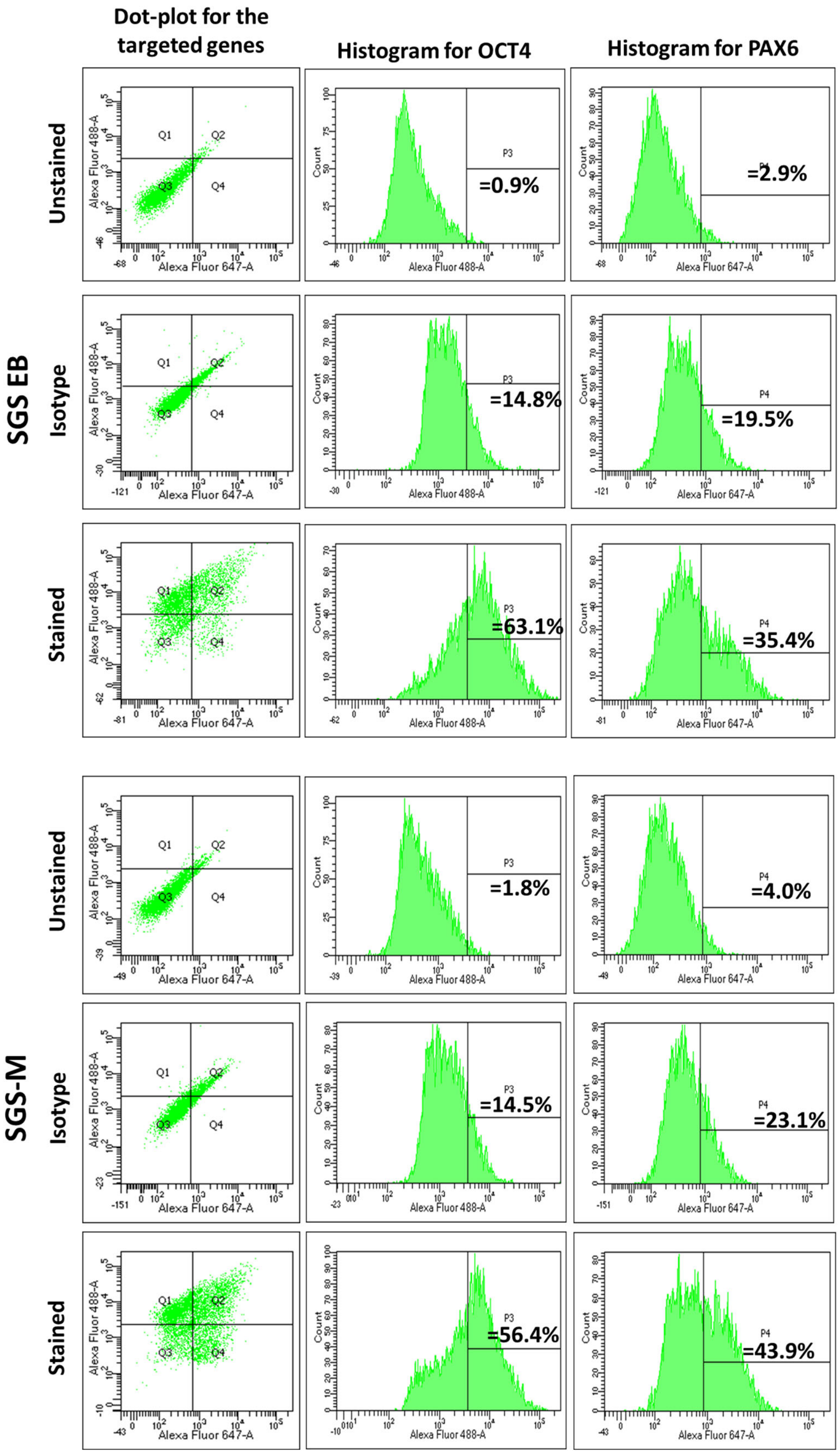
Dot-plot for the targeted genes
Histogram for OCT4
Histogram for PAX6
SGS EB
Unstained
Isotype
Stained
SGS-M
Unstained
Isotype
Stained
Q1
Q2
Q3
Q4
Alexa Fluor 488-A
Alexa Fluor 647-A
Count
P3
P4
=0.9%
=2.9%
=14.8%
=19.5%
=63.1%
=35.4%
=1.8%
=4.0%
=14.5%
=23.1%
=56.4%
=43.9%

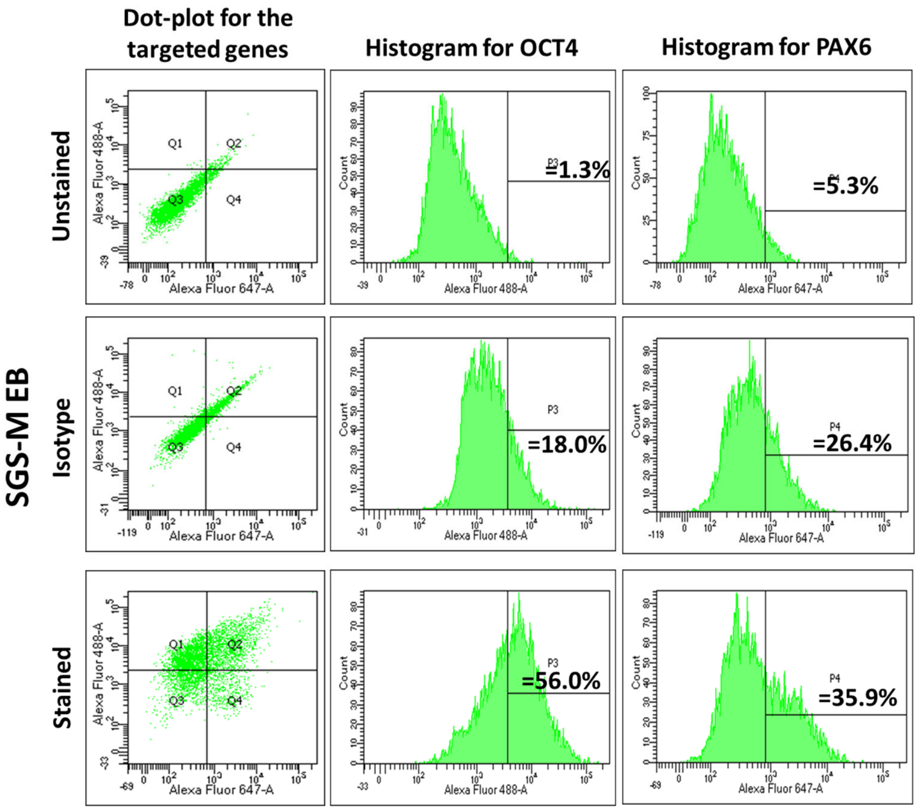

(d)

Figure S3. SGS matrix without EB induction showed a viable scaffold for ectoderm differentiation of hiPSC in 3D embedded condition. hiPSC was ectoderm differentiated in 3D embedded conditions in SGS and SGS-M, with and without EB induction. Cells were harvested on day 7. (**a**) Cell morphology of ectoderm differentiated hiPSC. The scale bar is 100 μm for 5X resolution and 50 μm for 10X resolution; (**b**) Multichannel images of immunostained control and ectoderm differentiated hiPSC. The cell was stained for nucleus, *OCT4*, and *PAX6*. Nucleus, *OCT4*, and *PAX6* were pseudo-colored with green, blue, and red respectively. The scale bar is 100 μm and the resolution is 10X; (**c**) Montage view of Z-stack multichannel images of immunostained control and ectoderm differentiated hiPSC. Cells were stained for nucleus, *OCT4*, and *PAX6*. Nucleus, *OCT4*, and *PAX6* were pseudo-colored with green, blue, and red respectively. The images present merged channels. The scale bar is 50 μm and the resolution is 20X; (**d**) Flow cytometric analysis of control and ectoderm differentiated hiPSC for *OCT4* and *PAX6*. *OCT4* was labeled with AF488, and *PAX6* was labeled with AF647 fluorochromes. Each sample was analyzed as unstained for autofluorescence, isotype for nonspecific binding, and stained for specific binding.
